# Bilateral Atypical Facial Pain Caused by Eagle's Syndrome

**DOI:** 10.1155/2020/3013029

**Published:** 2020-02-25

**Authors:** V. Anuradha, Ravi Sachidananda, Satish Kumaran Pugazhendi, Preeti Satish, Romir Navaneetham

**Affiliations:** ^1^HOSMAT Hospital, Magrath Road, Richmond Road, Bangalore 560025, India; ^2^Department of Oral and Maxillofacial Surgery, M R Ambedkar Dental College and Hospital, Bangalore, India; ^3^People Tree Hospital, Tumkur Road, Goraguntepalya, Bangalore 560022, India; ^4^Annasawmy Mudaliar General Hospital, Pulkeshi Nagar, Bangalore 560005, India; ^5^Oral and Maxillofacial Surgery, Vydehi Dental College, EPIP Area, Whitefield, Bangalore 560066, India

## Abstract

Recurrent throat pain, “foreign body” sensation, difficulty in swallowing, or vague facial pain is many times caused by the presence of an elongated styloid process. Many times, this condition is misdiagnosed and the patient is treated for facial neuralgia. But once Eagle's syndrome is confirmed by clinical and radiological examination, the treatment is always surgical resection. The approach maybe intraoral or extraoral. In this paper, we present a case of Eagle's syndrome caused by bilateral elongation of the styloid process and where surgical resection of the same gave instant permanent relief for the patient.

## 1. Introduction

One of the rarer conditions causing chronic craniofacial pain is Eagle's syndrome. This was first described by Watt W. Eagle in 1937 [1]. Many times the vague nature of presentation and quality of the pain serves to muddy the waters and leads to the patient being seen by many consultants like the neurosurgeon; the ear, nose, and throat surgeon; and many times even by a psychiatrist. Therefore, it is very common for these patients to be diagnosed with idiopathic nonspecific facial pain and to be treated without any positive result or relief from pain [2, 3].

The normal styloid process is a part of the temporal bone and usually measures about 25 mm in length. Clinically, discomfort is seen in patients where the elongated styloid processes are 40 mm or longer. Presenting symptoms include intermittent facial pain, sore-throat-like symptoms, ear pain, “foreign body” in throat sensation, or vague cervical pain [3, 4].

Eagle's syndrome is caused by a lengthened styloid process or by the links of the stylohyoid ligament getting ossified like a chain. Because of this, Eagle's syndrome is also sometimes called the stylohyoid syndrome [5]. This elongation of the styloid process maybe unilateral or bilateral. It is important to note that many times this abnormality is found only during routine radiographic examination without any presenting clinical symptoms [6]. In these patients, no active intervention is necessary and the presence of an elongated styloid process may be treated as an anatomical variation.

The normal styloid process is angulated downward, forward, and medially from the temporal bone. The tip of the process is situated between the internal and external carotid arteries and posterior to the tonsillar fossa and lateral to the wall of the pharynx. It is here that many times the tip can be felt intraorally. The muscular attachments of the styloid process is the stylopharyngeus, stylohyoid, and styloglossus. There are also two ligaments attached to the process, the stylohyoid and stylomandibular ligaments [7].

The pain is caused by (1) pressure on the glossopharyngeal nerve or trigeminal nerve or the chorda tympani branch of the facial nerve; (2) compression of adjacent structures; (3) compression of the sympathetic chain on the carotid sheath; (4) tendonitis of the stylohyoid muscle; (5) pressure on the mucosa covering the pharynx; (5) rarely, neuropraxia of cranial nerves 5, 7, 9, and 10 after tonsillectomy; (6) rarely, death due to vagus-mediated cardiac arrest; and (7) pressure on the internal carotid artery causing a transient ischemic attack [3, 8].

Commonly, routine orthopantamographs or computed tomography scans reveal the elongation of the styloid process. There is a classification that strives to describe the elongated styloid process radiographically [9] (Table 1).

The only accepted permanent treatment protocol for this anatomical abnormality is surgical removal of the elongated styloid process.

## 2. Case Presentation

**Figure 1:**
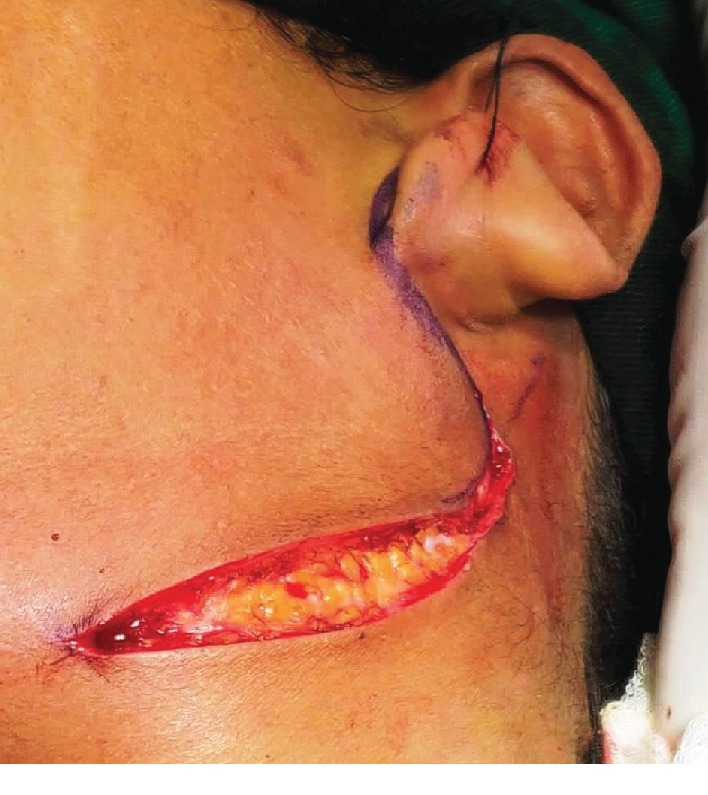
Incision.

**Figure 2:**
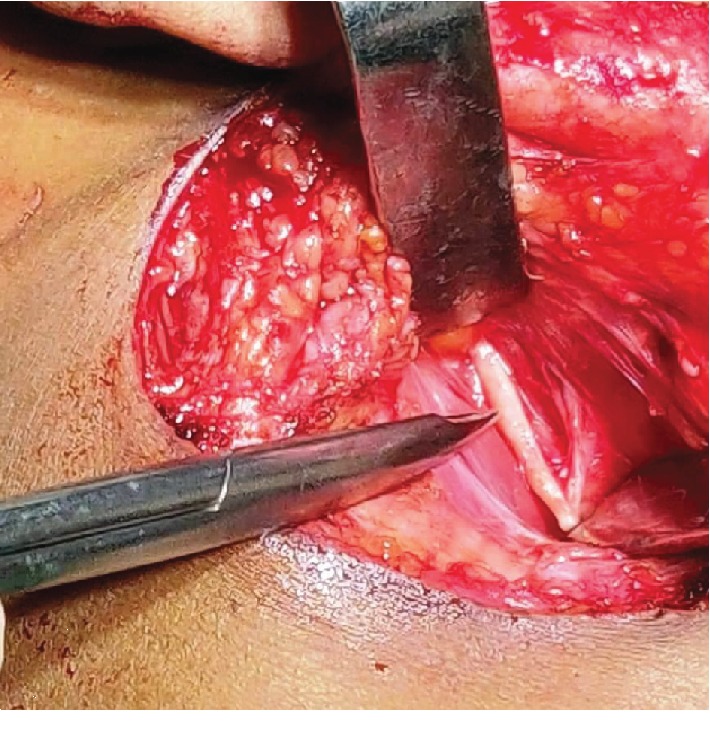
Right-side styloid process (Type II).

**Figure 3:**
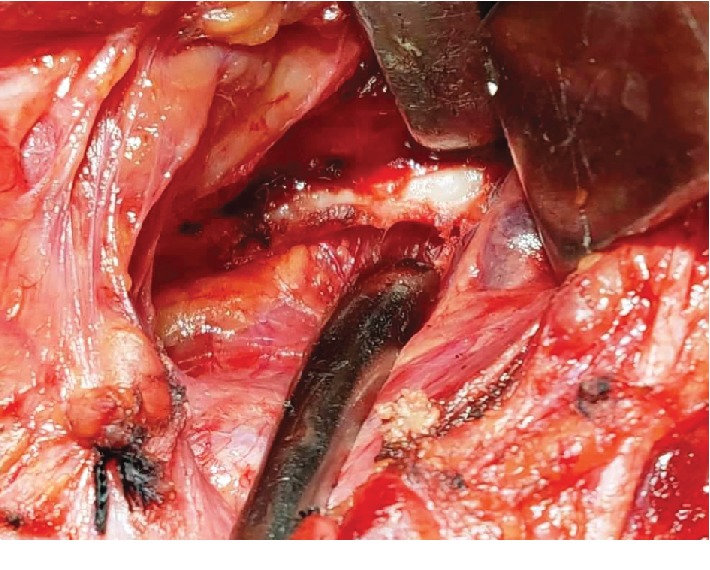
Left-side styloid process (Type I).

**Figure 4:**
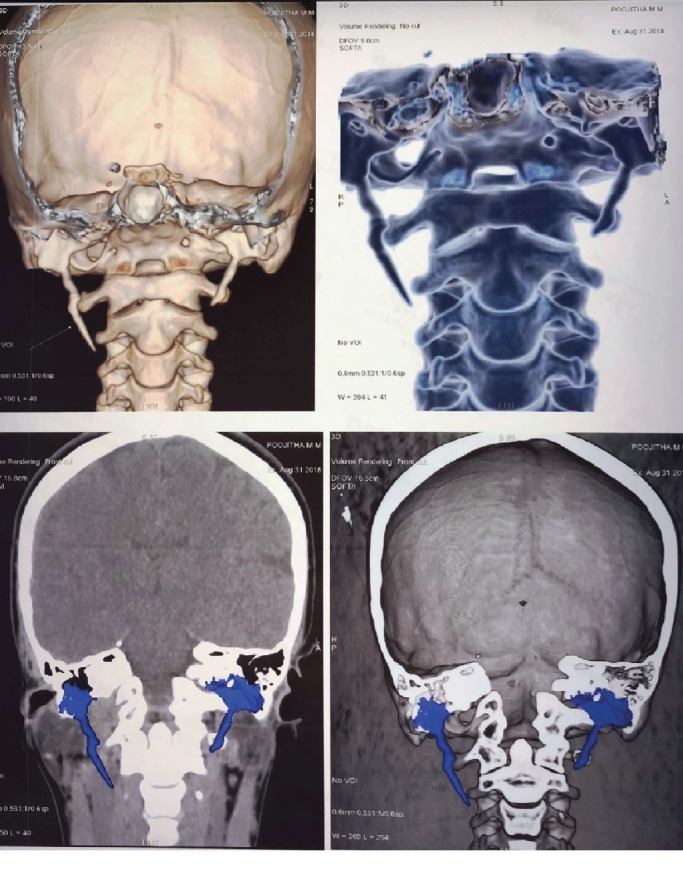
Computed tomography and 3D reconstruction.

**Figure 5:**
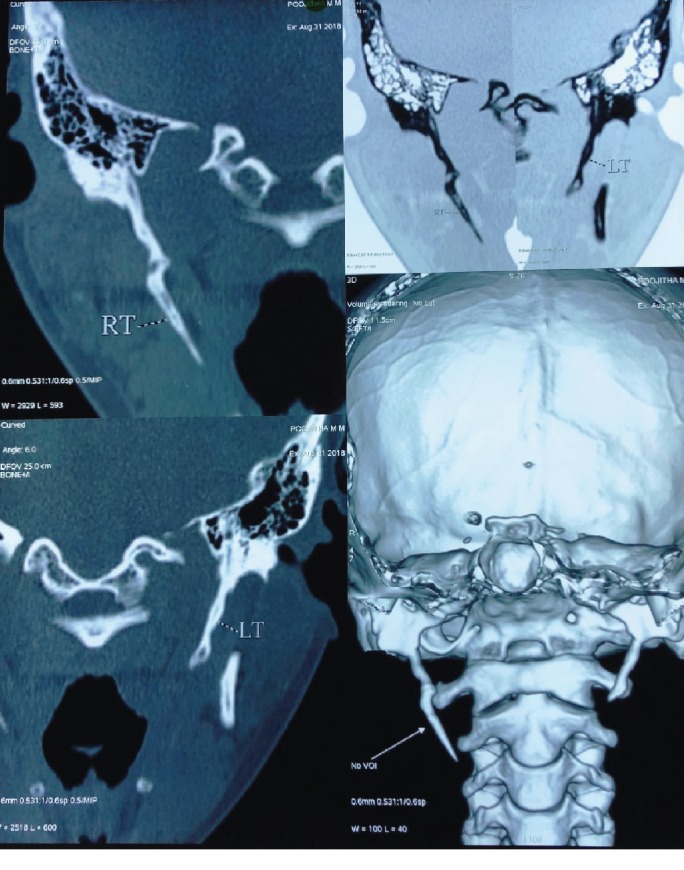
Computed tomography and 3D reconstruction.

A thirty-two-year-old female patient presented to the HOSMAT hospital with a complaint of bilateral neck pain which was exacerbated when the head was turned to either side and during swallowing since 4 years. She had been reviewed by many consultants over the past few years for the same without getting any relief. She was referred to us by the Ear, Nose, and Throat Team. There were no other clinical symptoms like headache, temporomandibular pain, or ear pain. There was no history of previous trauma or any surgical procedure. On palpation, the styloid process could be felt quite easily intraorally and extraorally. A course of oral amitriptyline was offered to the patient in an attempt to treat the condition conservatively, but this did not help the patient get relief from pain. Therefore, surgical excision via Risdon's incision (Figure 1) was planned for the bilaterally elongated styloid processes. The patient was offered surgical excision through an intraoral approach, but declined the same when advised of the need for elective tonsillectomy bilaterally. When the styloid process was evaluated radiographically, on the right side it was found to be a Type II variation and on the left side a Type I variation. This was confirmed during the intraoperative phase (Figures 2 and 3). Investigations performed included computed tomography scan with 3 dimensional reconstruction (Figures 4 and 5).

**Figure 6:**
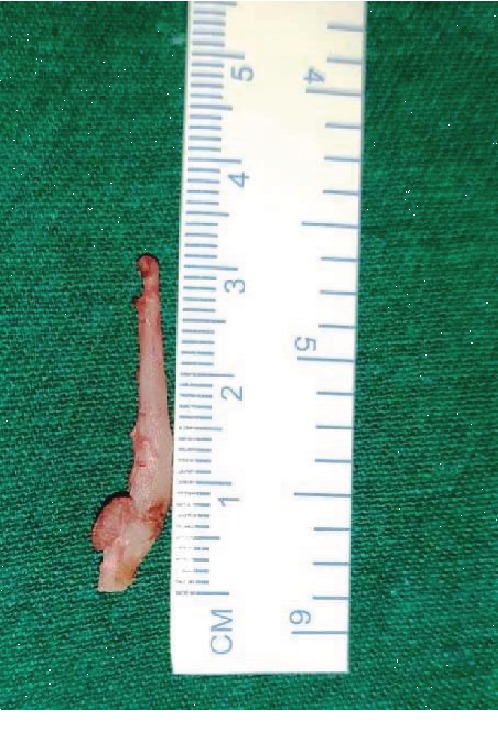
Excised right styloid process.

**Figure 7:**
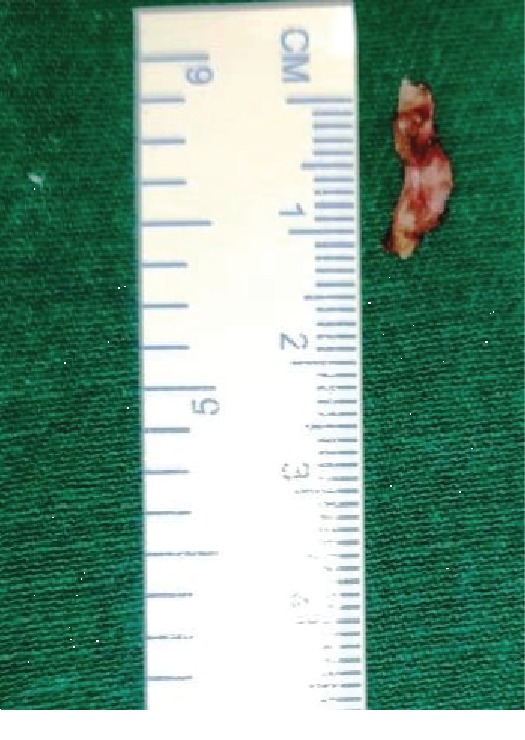
Excised left styloid process.

**Figure 8:**
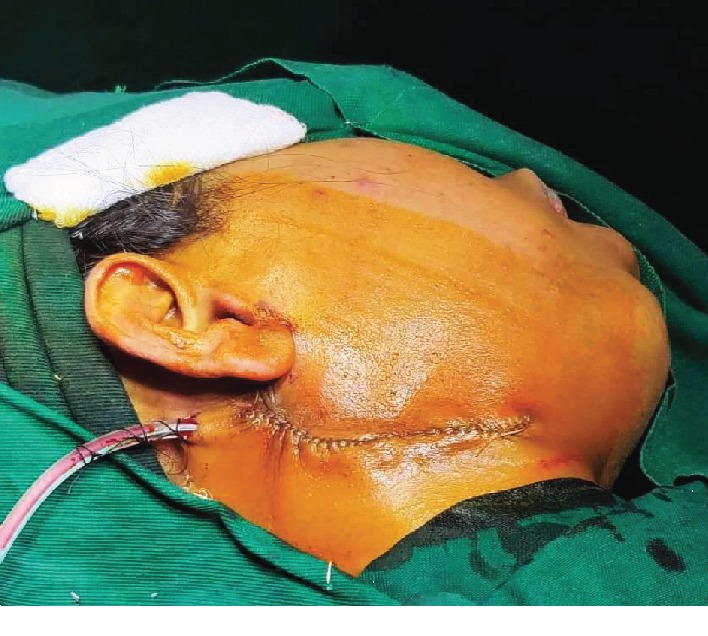
Closure.

The resection of the elongated styloid process was done with general anesthesia with oral intubation. After Risdon's incision, a subplatysmal flap was elevated with care to preserve the marginal mandibular branch of the facial nerve. The posterior border to the sternocleidomatoid muscle and the posterior belly of the digastric muscle was identified carefully, and the dissection was done in the stylopharnygeal recess. The tip of the elongated styloid process was easily felt in this gap. The entire length was exposed with judicious use of monopolar and bipolar diathermy. The tissues underneath the styloid process were protected using retractors, and the dissection was carefully extended cranially to enable a lengthier resection. A 701 bur was used to cut the elongated styloid process (Figures 6 and 7). Plastic closure with drains in situ was employed (Figure 8). When the patient was reviewed after 24 hours, she was totally symptom-free. Now at one year after surgery, she continues to be symptom-free and is free of any medication.

## 3. Conclusion

A thorough knowledge of the pain symptoms caused by the elongated styloid process is very important for the oral and maxillofacial surgeon. Eagle's syndrome must always be considered in the differential diagnosis of patients with cervicofacial pain. Once the diagnosis is confirmed by clinical and regional imaging, resection of the styloid process is the treatment of choice. The immediate relief from pain and other symptoms of the patient is indeed a justification for the surgical resection of the elongated styloid process.

## Figures and Tables

**Table 1:** Classification of the elongated styloid process.

Type I: a single-piece elongated process
Type II: a union of the stylohyoid ligament and stylohyoid process by a pseudoarticulation which is single
Type III: consists of multiple segments of the ossified stylohyoid ligament with multiple pseudoarticulations
